# Experiences of seeking and receiving maternity care during a health-system shock: a qualitative study with women and partners in the UK with a focus on marginalised groups

**DOI:** 10.1136/bmjph-2025-004303

**Published:** 2026-03-27

**Authors:** Tisha Dasgupta, Lara Rowell, Harriet Boulding, Abigail Easter, Gillian Horgan, Hiten D Mistry, Yesmin Begum, Michelle Peter, Tania Sutedja, Aricca Van Citters, Eugene C Nelson, Peter von Dadelszen, on behalf of The RESILIENT Study Group, Laura A Magee, Hannah Rayment-Jones, Sergio A Silverio, Laura A Magee

**Affiliations:** 1 Department of Women & Children’s Health, King’s College London, London, UK; 2 GKT School of Medical Education, King’s College London, London, UK; 3 King's College London King’s Policy Institute, London, UK; 4 Department of Population Health Sciences, University of Leicester, Leicester, UK; 5 The ENGAGE Study PPIE Group, London, UK; 6 The RESILIENT Study PPIE Group, London, UK; 7 The Dartmouth Institute for Health Policy & Clinical Practice, Dartmouth College, Hanover, New Hampshire, USA; 8 Department of Psychology, University of Liverpool, Liverpool, UK

**Keywords:** COVID-19, Qualitative Research, Sociodemographic Factors, Health Services Accessibility, Public Health

## Abstract

**Introduction:**

Health-system shocks, such as the COVID-19 pandemic, significantly disrupted routine maternity care. This study explored the lived experiences of accessing maternity care during such a shock, with a focus on marginalised populations and those with social or medical complexity.

**Methods:**

Semi-structured interviews (n=55) were conducted with 40 women and 15 partners across the UK who accessed maternity care during the pandemic. Data were analysed using Template Analysis, guided by an extended Candidacy framework, which explores how healthcare eligibility is negotiated between individuals and health systems.

**Results:**

At the individual level, limited information and disrupted relationships made it harder for individuals to assert their claim to candidacy, when seeking care. At the system level, services became harder to use due to new barriers associated with virtual care delivery and restrictions on partners. Judgements were made by professionals on cultural differences, birth plans and women’s opinions being disrespected. At the joint individual-system level, navigating the system was harder due to ever-changing restrictions. Service users resisted offers of care that did not align with their needs. Local conditions such as media messaging and heavy strain on the National Health Service workforce further exacerbated negative experiences of care. These barriers were exacerbated for those belonging to marginalised groups.

**Conclusions:**

Barriers to seeking maternity care during health-system shocks are layered across individual, system and joint levels, and disproportionately affect those facing social or medical disadvantage. Responsive policies and service designs must address these intersecting challenges to improve equity in access and care during future disruptions.

**WHAT IS ALREADY KNOWN ON THIS TOPIC**

- During the COVID-19 pandemic, maternity services in the UK were rapidly reconfigured, including increased use of virtual care and restrictions on partner involvement. Evidence suggests these changes negatively affected women’s experiences, but there is limited understanding of how people negotiated access to care during this period, particularly those facing social or medical disadvantage.

**WHAT THIS STUDY ADDS**

- This study demonstrates that access to maternity care during a health-system shock is shaped by ongoing negotiation between service users and the health system, rather than by service availability alone. It shows how women and partners actively worked to assert, resist or bypass care in response to disrupted relationships, inconsistent guidance and perceived judgement, with these challenges compounding existing forms of marginalisation.

**HOW THIS STUDY MIGHT AFFECT RESEARCH, PRACTICE OR POLICY**

- The findings suggest that emergency maternity care planning must consider not only service delivery models, but also how policies and professional practices affect people’s ability to claim care. Embedding partner involvement, culturally competent communication and adaptable pathways for virtual and in-person care may help prevent the widening of inequalities during future health-system shocks.

## Introduction

 In the UK, there are a minimum of nine antenatal care (ANC) visits recommended, with 12 recommended for primiparous women. ANC visits allow for exchange of essential information, establishment of a trusting relationship between women and healthcare professionals (HCPs), risk assessment and management of potential pregnancy complications.^1 2^ It is essential to the health and well-being of mothers and babies that ANC attendance is timely and experienced positively. Positive care experiences (such as access to information, involvement in decision-making over care and birth, feeling listened to and confidence in care providers) are associated with better pregnancy outcomes.^3^

During the pandemic health-system shock, the UK National Health Service (NHS) implemented substantial maternity service reconfiguration: fewer ANC appointments; a shift of some ANC visits to virtual delivery^4^,^6^; restrictions on fathers, partners and non-gestational parents from attending ANC appointments and sometimes, labour and the postpartum ward,^7^,^9^ as well as limitations on choice of place and mode of birth.^10^ Additionally, there was widespread uncertainty around rapidly changing guidelines, including the risk to pregnant women and the advisability of COVID-19 vaccination when it became available^11 12^; along with strict ‘stay at home and protect the NHS’ advertisements on social media platforms.^13^ As such, there was a contemporaneous reduction in ANC attendance,^14^ increased mental health concerns,^15 16^ higher rates of stillbirth^17 18^ and poor experiences of maternity care in general.^19 20^

Most research on maternity care-seeking has measured utilisation of services, without evaluating experiences of care and the potential impact on care-seeking of social, cultural and economic factors.^21^,^23^ The theoretical construct of ‘candidacy’ described by seven factors considers the decision to seek care as a process of continual joint negotiation between service-users and service-providers, in the context of the wider health system.^24^ Successful care access requires considerable work for service-users, who are likely to be deterred by difficult and complex access,^22 24^ or when there is a lack of alignment between expectations of care and provision of that care. ^22 24^A qualitative systematic review by our team of 51 studies (1347 participants) describing marginalised women’s care-seeking during pregnancy in high-income countries brought forth two additional themes of ‘intercultural dissonance’ and ‘hostile bureaucracy’ to capture the experiences of migrants (including refugees and asylum seekers), who face greater and unique barriers to care engagement in host countries.^25^

Using the theoretical lens of an expanded Candidacy framework, we sought to explore the lived experiences of care-seeking and care-receipt, of women and partners who utilised maternity care services during the pandemic, as a model of a health system shock, and with a focus on those from marginalised groups or experiencing social or medical complexity.

## Methods

Full details of the qualitative methodologies in the RESILIENT study have been published.^26^

In brief, women, partners, HCPs and policy-makers were recruited using a random purposive sampling strategy for in-depth interviews (IDIs) using a semistructured guide (see online supplemental file 1), conducted between May 2022 and February 2023 (by TD and HB both female researchers experienced in qualitative research). This paper presents data related to women and partners; interviews with HCPs and policymakers will be analysed and published separately. Interested individuals were asked to contact the research team directly, who assessed their eligibility; none were known to the research team. All who expressed interest were deemed eligible, and as such, no one was approached by the research team and there were no refusals. Those who were pregnant or who had partners who were pregnant or postpartum in the UK, and who utilised NHS maternity care during the pandemic, were eligible. Women and partners were interviewed separately; partners interviewed in this study were not the partners of the women who participated. Targeted sampling criteria were used to achieve diversity across ethnic minorities, deprivation status, geographic areas across the UK, and social and medical complexity. We sought to recruit a sample that was representative of the population of the UK, rather than solely individuals from marginalised groups. Social complexity was self-identified by participants and included: lack of social support, mental health problems or belonging to a minority group relating to sexual orientation or gender identity. Medical complexity was defined as having had to perform self-monitoring of symptoms during pregnancy for any complication, including hypertension, gestational diabetes, additional scans for predisposition to genetic complications or previous pregnancy loss. We sought information on participants’ lived experiences during the pandemic health-system shock, specifically: seeking maternity care, receiving said care, support from partners, availability of information and adapting to rapidly changing guidelines. Recruitment ceased once sufficient diversity across the prespecified participant characteristics had been achieved, and as such in expected lived experience.

Transcripts were analysed using Template Analysis^27^ methodology, according to the extended version of the Candidacy framework^25^ as the initial coding template (table 1) on NVivo qualitative research software. This theoretical framework, designed specifically for marginalised groups, describes how the eligibility and consequently use of healthcare services is continually and jointly negotiated between the individual and the healthcare system.^24^ This extension consisted of an additional two factors to the Candidacy framework’s original seven,^24^ which have been classified as individual-level, health-system-level or joint-level factors. However, interview topic guides did not directly ask about participants’ immigration history or its impact on their pregnancy, thereby resulting in limited data pertaining to these constructs.

### Patient and public involvement

The RESILIENT Study benefitted from a Patient and Public Involvement and Engagement Advisory Group (PPIE-AG; n=15) and a Technical Advisory Group (n=19), both UK-wide, who periodically reviewed data analysis and interpretation. Named PPIE members (TS, YB and MP) were involved in validating findings and reviewing this manuscript.

**Table 1:** Constructs of an extended Candidacy framework^24 25^

Candidacy framework factor	Definition	Individual level	Health system level
Existing factors			
Identification	Self-acknowledgement of necessity of medical attention for symptoms	●	–
Navigation	Learning about and negotiating services	●	●
Permeability	Ease with which people can use services	–	●
Appearance at health services	Individuals’ ability to articulate their need for care and assert their candidacy	●	–
Adjudication	Healthcare providers’ judgements dictating progression of individuals’ candidacy	–	●
Offers and resistance	Declining to accept care offers, medications or referrals	●	●
Local production of candidacy	Local factors influencing candidacy, including availability of resources and long-term patient–provider relationships	●	●
Extended factors proposed			
Intercultural dissonance	Additional barriers faced by those who are not native-born and experience a distinct difference in social norms and culture, medical and social knowledge and expectations, language, changing intergenerational relationships.	●	–
Hostile bureaucracy	Discriminatory policies in the host country, both in and outside of healthcare, that impose additional barriers such as restrictions on health coverage, welfare support and right to rental properties; high visa application costs; and limits on qualifying employment.	–	●

## Results

Semistructured IDIs (n=55) were conducted with women (n=40) and partners (n=15); table 2 presents participant characteristics. The median age of participants was 36 years (range of 23–44 years). Most identified as female (n=41; 74.5%) and were heterosexual (n=47, 85.5%), but there was representation from bisexual, lesbian and gay participants. Most participants (n=31; 56%), self-identified as White or White British, with 11% as Asian or Asian British, 18% as Black or Black British, 11% as Mixed or multiple ethnicities, 4% as any other ethnicity, and 1% who preferred not to disclose their ethnicity. While over 50% of the participants identified as White/White British, our study sample overrepresented minority ethnicity groups as compared with the national census.^28^ Almost half (n=26; 47%) of women and partners utilised maternity services in London, with 38% accessing those services elsewhere in England, and between 4% and 6% in each of Wales, Scotland and Northern Ireland. among women and partners, 25% reported at least one aspect of social complexity, and 44% reported medical complexity which required self-monitoring. Only one participant reported not having English as their first language. Care was provided by midwife-led or obstetrician-led teams.

**Table 2:** Participant characteristics

	Women N=40 (%)	Partners N=15 (%)	Total N=55 (%)
Age at Interview			
18–25	2 (5)	0 (0)	2 (3.6)
26–30	5 (12.5)	6 (40)	11 (20)
31–40	28 (70)	7 (46.7)	35 (63.7)
41–50	5 (12.5)	2 (13.3)	7 (12.7)
Gender			
Female	40 (100)	1 (6.7)	41 (74.5)
Male	0 (0)	14 (93.3)	14 (24.5)
Sexual orientation			
Heterosexual	36 (90)	11 (73.3)	47 (85.5)
Bisexual	3 (7.5)	3 (20)	6 (10.9)
Lesbian	1 (2.5)	0 (0)	1 (1.8)
Gay	0 (0)	1 (6.7)	1 (1.8)
Ethnicity			
White/White British	24 (60)	7 (46.7)	31 (56.4)
English/Welsh/Scottish/Northern Irish/British	19	5	
Irish	1	0	
Any other White background	4	2	
Asian/Asian British	6 (15)	0 (0)	6 (10.9)
Any other Asian background	0	0	
Bangladeshi	2	0	
Chinese	1	0	
Indian	3	0	
Black/Black British	6 (15)	4 (26.7)	10 (18.2)
African	1	2	
Any other Black/African/Caribbean background	2	1	
Caribbean	3	1	
Mixed/multiple ethnicity	2 (5)	4 (26.7)	6 (10.9)
Any other mixed/multiple ethnic background	0	0	
White and Asian	1	1	
White and Black African	0	1	
White and Black Caribbean	1	2	
Other ethnic group	2 (5)	0 (0)	2 (3.6)
Any other ethnic group	1	0	
Arab	1	0	
Geographic region			
London	18 (45)	8 (53.3)	26 (47.2)
East of England	2 (5)	1 (6.7)	3 (5.5)
Midlands	3 (7.5)	0 (0)	3 (5.5)
Northeast	4 (10)	2 (13.3)	6 (10.9)
Northwest	3 (7.5)	1 (6.7)	4 (7.2)
Southeast	2 (5)	0 (0)	2 (3.6)
Southwest	2 (5)	1 (6.7)	3 (5.5)
Wales	3 (7.5)	0 (0)	3 (5.5)
Scotland	2 (5)	1 (6.7)	3 (5.5)
Northern Ireland	1 (2.5)	1 (6.7)	2 (3.6)
Index of Multiple Deprivation			
1 (most deprived)	3 (7.5)	0 (0)	3 (5.5)
2	3 (7.5)	1 (6.7)	4 (7.2)
3	2 (5)	0 (0)	2 (3.6)
4	4 (10)	1 (6.7)	5 (9.1)
5	5 (12.5)	5 (33.3)	10 (18.2)
6	5 (12.5)	0 (0)	5 (9.1)
7	4 (10)	4 (26.7)	8 (14.5)
8	6 (15)	3 (20)	9 (16.4)
9	5 (12.5)	0 (0)	5 (9.1)
10 (least deprived)	3 (7.5)	0 (0)	3 (5.5)
Prefer not to say	0 (0)	1 (6.7)	1 (1.8)
Social complexity			
Yes	14 (35)	0 (0)	14 (25.5)
No	25 (62.5)	15 (100)	40 (72.7)
Prefer not to say	1 (2.5)	0 (0)	1 (1.8)
Self-monitoring of symptoms*			
Yes	20 (50)	4 (26.7)	24 (43.6)
No	20 (50)	11 (73.3)	31 (56.4)
Care provided by*			
Midwifery led	21 (52.5)	11 (73.3)	32 (58.2)
Consultant led	19 (47.5)	4 (26.7)	23 (41.8)
Personal/household high-risk status for COVID-19			
Yes	8 (20)	4 (26.7)	12 (21.8)
No	32 (80)	11 (73.3)	43 (78.2)

* For partners, care provided by and self-monitoring of symptoms refers to their birthing partners’ use of services.

Qualitative results are presented below, with participant quotations supporting each of the 7+2 constructs of the extended Candidacy framework presented in tables3^5^: women’s quotations indicated by the prefix ‘W’ and partners with prefix ‘P’, and each having a unique numerical suffix.

**Table 3:** Candidacy framework—individual constructs

Identification of candidacy	Appearances at health services	Intercultural dissonance
There was a lot of misinformation. I didn’t feel clued up at all and I’m a very clued up person. I do research that stuff and I do ask questions. And yes, nothing was clear at all. – W002 I felt like what if my newborn baby just comes and then if he or she gets in contact with Covid, I was actually scared. But then I knew there was nothing I could do about it so all I could do was to make sure that I prevent her, she didn’t step out—that’s my wife—she didn’t step out anywhere. All her medical appointments were actually online, and sometimes the doctor came in to our house, and whenever the doctor came in I made sure that she sanitised her hands and she was with her nose mask and all of that, I made sure that she took the Covid-19COVID-19 guidelines very serious too in order not to infect my wife. – P002 There were a couple of over the phone appointments rather than going in to see my midwife. I was okay with that as well. I was slightly concerned that if I wasn’t seeing someone in real life maybe they would miss something or there might be something that they monitor, like they might have taken a urine sample or something like that. – W002 I did have a sort of like low level of anxiety about what if there is something wrong? Like, it was nothing very apparent that she was having, you know, she wasn't very ill, it was more about would she develop properly? When she gets older, what does this mean? We had a Teams meeting when she was, I think, 8 months old. Said that everything was going fine, and she seemed to be developing normally. Since then, it turns out, and I don’t know if this is related to the brain structure, she was very late to meet all of her physical milestones, and they've since discovered that she has a chromosomal anomaly. And when they looked back at it, she was late to meet all of those physical milestones. So, it’s possible that it would have been picked up sooner if more people had seen her. – W034 I’d had some miscarriages before I had my first, I had early scans with him. None of that was running. We managed to find somewhere privately. – W028 Having said it’s very difficult to do calls with health visitors over the phone, I felt like the support… I was able to reach out for a lactation consultant and things like that. And they did some great digital online support and things. So I felt like when there were issues, I was able to then source support. But there are a couple of factors that go into that, you have to be able to pay for it, which I was fortunate enough to be in a position where I could pay for it, I was mindful that a lot of people wouldn't have been able to afford the costs. And also you have to sort of know enough about what’s not working and about what you're sort of worried about in order to then reach the right care, if that makes sense, and you have to do it on your own. So it’s I think, again, that, sort of, barrier to access then for some people, that would have been too much to overcome. – W039 I just searched the internet and came across a charity that tries to make improvements and things for people that have suffered previous stillbirth, miscarriage. Yes, so I did some research on them and came across a professor who works at the [specialist clinic for women following stillbirth], and he’s a professor that specialises in stillbirth, stillbirth prevention, whatever, he’s done lots and lots of studies, I think he’s quite known worldwide for what he’s done. So we got in contact with them, because my husband was like, “Oh, it’s a no brainer, we’ve got to do it, we can’t know about that and then not do it. It doesn’t matter how far it is to travel.” So yes, I spoke to them I think via email to start with, got hold of his secretary. But I had to be referred from my GP, so I did persist with that. – W007	But that had been moved to Zoom. So we had to try and manoeuvre a device with a camera on it to film our daughter trying to latch and then show it to the breastfeeding consultant. She was very good, but she wasn’t very computer literate and so that whole thing was quite fraught. – P010 I don’t really know but there are some people who are not really comfortable speaking with someone online; sometimes they feel like maybe they would have explained themselves better. So yes, I think that was her problem, she felt like she could have expressed herself better while she was meeting with the doctor online, she felt like if it was in person she would have been able to express herself better. – P002 Because now my English is understandable, I can understand, and what I’m saying they can understand as well. That’s why I didn’t feel hard that way with the communication, but it’s moral support for when I was pregnant, they said I need an operation because my placenta is low, that time my husband wasn’t with me, but that’s why I felt like if he was with me, he can hear what they’re saying, but I can’t hold everything, this kind of thing, he needs to hear, but he wasn’t there. – W036 I think that made me feel a bit lost, maybe, along the way. We communicate quite well, but it is different hearing things second hand. I wasn’t there, so I didn’t hear things from the GP practice. – P001 We did have checks. Again, largely because we fought for them. So, [baby] was tongue tied, which made breastfeeding difficult. He had silent reflux, which made feeding difficult. Wasn't putting on weight. And even though we had all of those markers, even though we had all of those risk factors, we still had to push fairly hard to get appointments and visitors and things like that, because again, there was this feeling of not wanting to come over unnecessarily, heavily under-resourced, restrictions, not enough bodies to come around to see people… Even though we did have access, we did have to fight, we did have to push, we did have to be insistent and annoying. And when we did so, we didn't just do it based on neurosis. – P007 But I knew that I should have seen a midwife by eight weeks, but I didn’t. That was all done on the phone. I had a feeling I was going to have problems with my blood pressure again as I did in my previous pregnancy, so I was a little bit worried about not seeing somebody until later on because of that. My first face-to-face with anybody was at 12 weeks after my 12-week scan, when I saw a midwife who did the bare minimum. There wasn’t any kind of examination; it was just going through a few questions, prescribing aspirin, based on my BMI and my previous medical history. But it was all a bit disjointed, and I don’t think I met my community midwife at all until about 16 weeks. – W024 I was just in pieces, in tears, because apart from my kid and my in-laws and my partner I hadn’t seen anybody. So I was going into these places and just breaking down and unable to [pause] [sigh]… Yes, just feeling very overwhelmed. Overwhelmed is a really good way of explaining it. They did get me in contact with the Mental Health Midwifery Team, and I had one phone-call on a day that had been quite nice, I think it was just as we were starting to break away from the really hard lockdown and I’d gone “Oh yes, we’ve been and bought ice-cream,” or something like that… And she’d phoned me up and I’d gone “Oh yes, we’ve had a great day, we’ve done this,” and she went “Oh well, you don’t need to speak to me again, do you?” And then three days later I was banging my head against a wall because I was so overwhelmed with what was going on. – W027	But my mother-in-law was not very helpful talking about breastfeeding. She used to just say, “Oh I had so much milk they used to choke on it,” that kind of thing which is not very helpful. – W021 Other than that, sometimes mums who don’t speak English, they can be rude to them, the nurses, and talk about them in English in front of them and call them lazy and things like that. That was just atrocious to listen to, honestly. – W033 The area that this midwife usually served in my local area is an area where it’s predominantly minority, well you say minority but Black Asian minority ethnicity… I did think that’s probably why she was how she was - just very blunt and matter-of-fact. There wasn’t any sort of… You know the nice language that medical staff wrap around horrible facts… she just had to be straightforward because of the language communication and the assumptions that I wouldn’t understand. – W025 But they did record ethnicity and information about both of us. So I’m from an Ashkenazi Jewish background and there are certain inherited health conditions which are more prevalent in that community, and I don’t know if they did any additional testing around that. – P010 But I couldn’t tell her anything, because I thought if I told her something rudely she’s not going to care about me. Inside I was scared because I thought, “My baby is not going to be born properly if I speak rude to her like this. They are not going to help me properly.” – W036

**Table 4:** Candidacy framework—health system-level constructs

Permeability of services	Adjudication	Hostile bureaucracy
During that appointment then it was really nice because it was like a video call. We could see her, she could see us. And so even though we couldn't see each other face to face, we were still able to get to know her and she asked us lots of questions as well. So again, it felt like we were in good hands. – W005 Yes, it makes me feel better because I would still communicate with our doctor and it made it easier for me because at that point I didn’t have to ask my wife to explain those things that the doctor asked her to do and those things that he asked her not to do, so it makes it easier for me that whenever I’m with her, I just remember or I just know that the doctor said she had to do this and she does not have to do this, so that makes it easier for me to take care of her. – P002 Yes, one of the problems of booking the virtual appointment was that I had to wait on a very long—like the waiting list was always very long. Sometimes they would be attending to someone else for a long time. – P006 I would say the part that didn’t really go well was that sometimes I had some connection issues while trying to connect with my doctor and I think that was a bit of distraction to me, to myself and my doctor. – W017 My husband was not allowed to go to any of the appointments, so because of what had happened previously with having a miscarriage, it was always a bit stressful going on my own. Also, because they were umming and ahing over whether I would have a planned c-section or a vaginal birth. I felt like I was under pressure to take in a lot of the information under stressful circumstances so that I could then discuss it with him when I got home, so that was really difficult. – W020 Obviously I wanted him to be there for the birth, but during labour, it was almost like I didn’t even really need him there. Obviously I do know that that’s important but it was the bit after that I felt was the most important. I needed him there overnight. And so that was really hard. And that led to a downfall in my mental health at the hospital. So yes, I ended up having a little, mini breakdown because of the lack of sleep and him not being there to support me and the situation that I was in, basically. – W002 I was fully prepared that it was going to be bad news and that’s what I expected to receive that day. So having to go to it alone was really, really difficult, to the point that I remember going into the scan and just completely falling apart, thinking, “I'm not going to hear a heartbeat here and how am I going to get back home?” And, you know, all of this, and I knew my husband was going to be sat at home really nervous. We hadn't told anybody else about it just because we didn’t really want to give anybody bad news for the third time. So we were just holding this and it just made no sense to think, “We just can’t. But what happens if this is bad news? Like, what do we do? And how am I going to deal with it?” So it didn't make sense. I thought it was really cruel. – W006	So, we passed the due date when she went into labour and then so we did the normal thing, phoned the helpline and they said ‘okay, stay at home until the contractions reach a certain pitch and tempo.’ And at that point we drove to the hospital and again, I had to wait in the car while my partner went in, had to walk in while having contractions. And then wasn’t admitted and sent out again and we went home. And we ended up going to the hospital three times before she was eventually admitted. And I think the reason that it took so many times is that the only thing that they were measuring was dilation. So my partner wasn’t dilating, even though her labour was progressing in other respects. So it was only the third time that we went in, when actually the shift pattern of the attending nurses had come back round and there was a nurse who had been there the first time, who realised actually, you shouldn’t still be at this point of the process. And then they started to look for other factors and then we were admitted quite quickly after that. – P010 Which, I didn’t think it was, but then she’s the midwife, so I’m listening to her and her advice… So I got sent home. When I got home, less than an hour and a half later, I had a massive bleed… and I couldn’t get out of the car, because I could feel I was bleeding. And I’d already started contractions on the way home, as soon as my waters had gone, but obviously they said, “Just carry on, you know when your contractions get however many minutes apart it is on the paperwork to come in.” … So I rang the maternity unit, and when I was talking to them, I was like, “I’ve got to get off the phone,” and they said, “Oh, ring 999,” because it became profuse. I ended up getting blue-lighted back to the same hospital, and this is all in the space of an hour and a half. And they confirmed that my daughter had died because of this massive bleed, so she was stillborn the next day at full-term. – W007 Nobody checked to make sure baby was latching right or anything, it was just, “You know what you are doing” and that kind of thing. Mind, I’ve just had a thought now about 2020 baby. I don’t know where that’s come from. She was quite sleepy for the first 3 days, and the midwife came out on day 1 after… I gave birth at half past 7 at night and she came out the next afternoon, about 3 o’clock, asked how feeding was going… I was like, “She’s not fed since she’s been born. She’d fed for an hour when she was born but she’s not been fed again.” And she were like, “Oh right. But she still seems really sleepy. Oh, you know what you’re doing.” And that was it. There was no offer of any help to try and get this baby to latch on or anything. – W015 So yeah, he just basically said, “Have you had both your vaccines?” And I said I’d had the first before I was pregnant and we’d decided, as a couple, that I didn’t want to have my second vaccine until after I’d given birth. I said that I had no problem against the vaccine, I fully supported the vaccine, just while I was pregnant, I wasn’t 100% comfortable having it. And she asked me why, and I just said that I don’t believe there’s enough research from women having… especially when I was over my due date already and I’d had Covid two weeks before… And she said, “There’s absolutely no reason why you shouldn’t have the vaccine, enough research has had it… there’s never been any problems with any pregnant women who has had the vaccine before.” They were in the hallway and doing like walk-ins, vaccines, so she sort of said, “We are doing walk-ins today, so you should just go and get your vaccine.” And I sort of said, “Alright, that’s fine.” So I just sort felt pressured into it, and then she’d contacted the people who was doing the walk-in vaccines and said that I was heading down. So I just sort of felt like I had to go through with it. – W010	We were at [hospital] so there were quite a lot of women who were being taken outside who did not speak English, they were not allowed to bring their husbands in or partners in and they were being given bad news. – W020 They didn’t listen to what I wanted to say. Because they didn’t care about me, because of my speaking, I think I am ignored in so many ways. Like when I was in hospital, when I was in the doctors, I feel not happy, “Oh, she’s not speaking English, she needs somebody to interpret,” or this kind of thing. When I was in hospital, I couldn’t speak English, they didn’t bring an interpreter for me as well, and my husband, he is not a clever man as well, he didn’t arrange for an interpreter for me, and he said, “Oh, when you need something, just call,” and when I called, they came screaming to me. My husband needed to arrange for an interpreter for me, but he didn’t, and the health visitor, nobody arranged anything for me like that. – W036 I’m also [Nationality] but I’m originally from [Country outside the UK], so my family is there… And then my family were actually due to arrive in the UK on I believe March 27th of 2020 and, of course, their flights were all cancelled and, yeah, they weren’t able to come. And so that was… yeah, that was really isolating. – W038 She (the nurse))came and she was so rude to me, and she said, “Why is your husband not coming? Call your husband to come and look after you and your baby. It’s not our job to look after you and baby.” At that time, I am speaking like when the pandemic was on, this baby, I said, “My husband can’t come because he can’t take my son with him. Who’s going to look after my son? That’s why he can’t come. He’s not allowed to come here as well. Why are you screaming like this? Why are you saying my husband needs to come here?” and she is saying, “Who is going to look after your baby, then? Tell your husband to come. You don’t have any other relatives?” she is saying she doesn’t have any responsibility; everything is I have to do, and my husband needs to do. – W036

**Table 5:** Candidacy framework—joint-level constructs

Navigation	Offers and resistance	Operating conditions and the local production of candidacy
I wasn’t expecting not to have a midwife. So I think at that booking appointment, which takes place around 10 or 11 weeks, there was, I think, nobody in the midwife role at my GP surgery. So if I had any questions or concerns, I didn't really have anywhere to go to. And I think whilst people did their best, I just sort of didn't really belong to anyone, and I think that’s why I think for some of the appointments I ended up going to different doctor’s surgeries that weren't my own to be seen by just, you know, somebody that was available. – W003 I suffered from hyperemesis during my pregnancy, which was severe vomiting. And at the time, at the beginning of my pregnancy, because lockdown was so strict, it was really, really hard to go into a GP or get an appointment. I know they had so many calls and people couldn’t get through. I remember I did this online consultation twice, which is something new that they started, online consultations, and the process was so difficult because the questionnaire is so long. – W033 I had a look on the [Hospital] website and there was a timeline as to the weeks and months at which I should be offered appointments. I was offered appointments at the exact same time, so I could follow that timeline and I felt peace of mind that, “Okay. I did anticipate this appointment next. I anticipate this appointment next.” I think the [Hospital] being so clear on that and also being clear on what would be covered in each appointment and what I needed to bring… made me feel more comfortable and confident turning up to the appointment knowing what was going to happen at that time. – W005 The main difference I noticed was actually the health visitors. I don’t think I’ve ever seen a health visitor for this baby. Maybe that’s incorrect but compared to what normally feels like quite a high level of intrusion. I don’t think—the health visitor seems like they just disappeared off the face of the earth. I think one of them phoned me at some point. – W014 And also the community midwife, she didn’t want to do anything. So I’d ask for a stretch and sweep… But they declined to do that, because they said the risk of face-to-face contact was too much, and it didn’t really seem to matter what we said, because I just felt really concerned, because obviously my bump wasn’t that big, and… but there was no real concern there, and it didn’t seem to matter what we asked for, our voices weren’t heard, nobody wanted to listen, nobody was really concerned. So I changed area completely. I went to another hospital trust, that’s about 45 minutes away from where we live. – W007 And so then all of my care during the pregnancy was done in [city], but then I did deliver the twins then locally, because obviously [city] is a three-plus hour drive. So there were concerns about getting to the hospital in time. And so it was then a sort of transfer of care, I guess, at the last minute when I then gave birth to the twins, to our more local facility. – W039 One thing that we found very difficult was not knowing what the restrictions would be at the hospital. And they seemed to change on a day-by-day basis. So, trying to find a way of finding that information. – W032 So for some appointments they were saying that women should come alone and then some of them they said that partners are allowed to midwife appointments but they have to stay outside and then they get called in. On the one where I did bring my partner, the midwife was a bit like upset that he was there and said that I should come alone. And then when I went on my own, they said ‘no, he can come’. So that was a bit confusing as to whether he had to be there at the midwife appointments or not. – W001	I think generally because we’d been through this loop a few times, we didn't really engage with the GP services because there’s no point. Just go straight to the midwives. – P007 I also had a run-in with a consultant who told me that I was having a planned C-section, and then who I—I think I’m a very good advocate for myself – and who I then said in the appointment “No, I’m not.” “No, no, no, I’m going to book you in,” and he was literally checking his diary. “I’m booking you in,” he said to me, and I was like “No you’re not because…” “But your baby’s small,” and I’m going “But I’ve had a small baby, my family babies are small,”… But I think I was able to advocate for myself to a way that I went out and spoke to the receptionist team and went “No, I’m not speaking to him again because he’s disregarding my feelings and my opinions.” - W027 It’s just the waste of the initial service they offered, which was this local family and health clinic. This woman had come round and wasn’t assessing him properly, she didn’t get to know him. That was more stressful because I was wasting my time with this woman who wasn’t offering anything. And then with set exercises for [name] to do, I'm like ‘He can do that’, but she didn’t really care. She was carrying these pieces of paper and that was her job done. – W019 I feel like because there was much hands off, it was a concern. ‘Are they doing it properly? If they are not going to sit in a room with me and talk through things, are they getting all the information that they need? Are they doing all the tests that they need to do?’ So, there was always that worry at the back of my head. Also, I am a clinical negligence solicitor, so I think I am always going to be a bit sceptical [laughter]. – W020 I’d said categorically I wouldn’t be induced again, because last time it went so wrongly. And I just felt like I had no control, it felt like all these things changing and all the rules that would come out for maternity, took all control away. So I actually, in the end, had an induction, because it felt like I had some control. It meant that my husband could join me, it meant that my son would be taken somewhere to be looked after, and that was planned… I needed something to grab onto and to be able to control, and so that’s why I ended up actually having an induction and not a planned caesarean. – W008 The confused messaging, the different messaging I got from numerous different healthcare professionals, it was completely not joined up. And then when it got joined up, it actually felt very scary to be saying no to the vaccine because everyone was kind of saying, “You must. It’s incredibly dangerous not to.” I felt very… almost press-ganged into having it and that if I didn’t, I was… look, women and birthing people are damned if they do and dammed if they don’t, right. – W009 And then I was told everything was fine but actually I’ve been doing private scans because of my anxiety and Maternity will only offer so many scans under the NHS. They then picked up an irregular heartbeat and recommended a 24hr tape, but it wasn’t the NHS or my Maternity team who did that, so I then had to go back and say, “Privately, they’ve recommended this”…Maternity don’t like me going privately for anything because then they say there’s too many cooks, which I understand but then I also feel like I don’t… It’s not I don’t trust the NHS. They are so stretched and so busy, I feel like they are not always on the A game or thinking outside the box or connecting the dots, that I feel the need to supplement privately because I get more time and that person is listening and that person is able to offer me more tests, quicker tests, quicker results. – W022	I was devastated at giving formula because that wasn't my plan, but also there were shortages. So, you were having to drive around supermarkets trying to find the formula that you could get. You couldn’t get anything. – W030 All of a sudden, despite having worked for twenty-eight weeks saying, ‘No, I am not vulnerable. I can do these things,’ the government splash it all over the news that pregnant women are vulnerable, you should not go out, you should stay in, do not infect your baby, do not infect yourself, it put a huge pressure on me because I was worried about me becoming ill, the baby becoming ill, making my husband ill, would he be able to come to appointments with me or not? So, the government were constantly saying, ‘You are a vulnerable group. You are a vulnerable group.’ I was terrified. I think we are all were. So, yeah, that heavily influenced everything. My family stayed well away from me because it was all over the news that pregnant women were put in this vulnerable group… I felt like it was, ‘Well, it was your choice to get pregnant, so you have put yourself in this situation.’ I felt like I was adding a burden to the NHS. – W020 I wanted to go and take him to mother and baby groups. I really wanted to use it as an opportunity to become part of a community in a way that I haven’t been able to before and it just didn’t happen. And it was so appallingly isolating, and I just felt like we were… I don’t know, we might as well have been wolves raising him, sometimes I felt like we just had no support, no understanding from… no other parents around. – W021 It was a great experience because I got the chance to actually take care of our baby, to be there whenever it cries, maybe when the mother is doing something else I can pamper him, take care of him, make him smile a bit, so I would say it was a lovely time because I spent some great time with the family. – P003

### Individual-level constructs

#### Identification of candidacy

Identifying the need for seeking maternity care during the pandemic was characterised by uncertainty and a lack of information. Partners were concerned about the well-being of both mother and baby but reported being helpless to act. Participants struggled to know when to assert their candidacy for care. Women reported not knowing when or how to identify signs of concern, both during pregnancy, as well as postnatally, or knowing when to request additional consultations. They cited concerns over missed opportunities to pick up complications due to lack of face-to-face visits. Participants who were able to afford it felt the need to resort to seeking private maternity care rather than relying on routine NHS care to alleviate concerns; the latter was deemed to be insufficient and of poor quality. This was particularly true in cases with previous pregnancy loss or complications. Some participants preferred to transfer their care to specialist clinics, far away from home, to feel reassured they were receiving the best care possible.

#### Appearance at health services

Asserting a claim to candidacy by sufficiently describing symptoms and the urgency for care was difficult during virtual appointments. Loss of body language in communication and lack of sufficient knowledge of English, as reported by one participant, exacerbated the problem; due to an over-reliance on using the correct words and medical terminology rather than relying on body language and movements. Similarly for partners, being kept out of appointments made them feel lost and unable to assert a claim. Participants highlighted the need to fight for their claim to candidacy, and having to repeatedly advocate for themselves and the care they wanted to receive. Reduced interaction with HCPs during this period highlighted the need for women and their partners to act as advocates. On the other hand, it was also easier for women to hide or downplay the extent of their poor mental health and need for intervention, if they were not visibly struggling on the day of the appointment.

#### Intercultural dissonance

The added construct of intercultural dissonance described additional barriers faced by women and partners who have a migrant background and may have different cultural norms, thereby finding it more difficult to navigate the host country’s health system and make a claim for candidacy. Differences between cultures and generational attitudes were negatively perceived by HCPs, and women felt they were treated differently based on their ethnicity. On the other hand, one partner was concerned their background had not been taken into consideration sufficiently. They also felt they could not speak up without being reprimanded or offered poorer quality of care.

### Health system-level constructs

#### Permeability of services

Maternity service reconfiguration due to pandemic circumstances led to removal of some barriers to access and institution of others. Virtual delivery of care was a positive experience for some women, making it easier to attend appointments, removing transportation costs and allowing partners to be an active participant in the appointment. However, for others, long wait times and poor internet connectivity made it harder to access care virtually. Restrictions on partners at all points of care caused a great deal of distress for women. Some were unable to comprehend all information provided and ask informative questions without a second person present. When partners were allowed to be present during labour and birth, most hospitals placed strict restrictions on visiting hours postpartum. Women had to handle taking care of their newborn baby alone, in a state of exhaustion and heightened emotions immediately after giving birth. They also had to deal with potentially receiving bad news alone during ultrasound appointments, which was particularly detrimental to those with a previous pregnancy loss.

#### Adjudication

HCPs’ judgements and disregard of women’s and partner’s opinions impacted experience of care and whether care was sought, sometimes leading to fatal outcomes. Others felt abandoned after birth when HCPs were unable to assess their need for continued support. Women also reported struggling with adjudications made about vaccination (against COVID-19) and receiving a dose while pregnant. These difficult conversations with HCPs led to some women feeling coerced into getting the vaccine, even though they had outstanding concerns.

#### Hostile bureaucracy

This construct relates to policies and bureaucratic procedures in the host country, both in and outside of healthcare, that impose additional barriers and are more likely to impact individuals with a migrant background. Some women who did not speak English (self-identified as a social complexity factor) well were not provided with interpreters, even when receiving upsetting news. Others felt the onus was on them to arrange for an interpreter or a family member who could speak English, although they were falling short. For those who had no family in the UK, immigration and travel restrictions during the pandemic proved particularly isolating, as family members were unable to be present for the birth or to help take care of the baby.

### Joint-level constructs

#### Navigation

Rapid reconfiguration of services during the pandemic made an already complex maternity care system more difficult to navigate, particularly for those with medical and social complexity. The quality of services and women’s experience of care were variable and largely dependent on the specific service and individual HCPs. Sometimes women were lucky enough to have a well-organised hospital in their area and thus had a positive experience navigating their maternity appointments. Some services simply stopped. When unable to receive the care they wanted, women were burdened to find a hospital which would provide better care, often leading to problems with transportation. Strict restrictions on involvement of partners at all points of care, along with ever-changing guidelines, led to confusion for many participants, particularly as they varied between different NHS Trust hospitals.

#### Offers and resistance

Negative experiences, both in previous and current (at the time of interview) pregnancies, caused women and partners to reject offers of care, or bypass specific services. Care was refused when perceived as a check-box exercise and a waste of time. During lockdown periods with strict infection control restrictions, women raised concerns over the quality of care offered. On the other hand, sometimes women were afraid to refuse offers of care for fear of judgement, and so they acquiesced, even though their feelings were disregarded. Women also resisted offers of care to regain control over their pregnancies which was largely lost during the pandemic due to restrictions and disjointed guidelines and messaging. When not in alignment with what they wanted, those women who were financially able to refused NHS care and sought private services.

#### Operating conditions and the local production of candidacy

Local operating conditions outside the health system influenced candidacy for and experience of care, both positively and negatively. The shortage of essentials in supermarkets and the associated media coverage led to anxiety for mothers who relied on formula to feed their baby. Vulnerability was forced on pregnant women, in the messaging of official guidelines and news reports. People were encouraged to only attend services if absolutely essential. Participants acutely felt the loss of the social side of pregnancy, as mother and baby groups were immediately shut down during the pandemic. Conversely, local regulations such as most employers encouraging working from home allowed some partners to be more present and spend time with the baby. Local allocation of resources, often vastly unequal between different areas of the UK, strongly impacted overall care experience and what services were offered to pregnant women, reducing their choice and control over care.

## Discussion

### Main findings

Our findings call for a multilevel approach to plan maternity care for future health-system shocks. This large qualitative study of women and partners’ experience of accessing and utilising maternity care during a health-system shock shows eligibility for healthcare is influenced by multiple overlapping complex factors at the individual, health system and joint level.

At the individual level, service users cited concerns over missed opportunities to identify complications due to the lack of in-person care. Loss of body language cues made it difficult for people to sufficiently describe their concerns. Both women and partners highlighted the increased need to advocate for themselves, particularly partners who were kept out of most appointments. Cultural differences were perceived negatively, and some women felt they could not advocate for themselves for fear of being reprimanded.

At the health system level, maternity service reconfigurations led to removal of some barriers to access and institution of others. Virtual care provided flexibility for some, but issues with using digital technology proved problematic for others. Without a birth partner present, women often had to receive bad news and process information alone and did not have any help caring for the newborn while in hospital. Provision of interpreters was also limited and difficult to achieve on remote consultations.

At the joint level, confusing guidance for pregnant women and rapidly changing hospital guidance made a complex system even harder to navigate. Service provision varied between different areas of the UK, reducing women’s choice and control over care. Consequently, many refused or resisted offers of care, choosing not to engage with specific services or seeking private services instead.

### Interpretation

The findings of this study are coherent with those of a systematic review conducted by our team on care-seeking during pregnancy among women with multiple social risk factors, in high-income countries globally, which found barriers to be centred around a loss of autonomy and dignity; lack of informed choice and decision-making; poor trust in and relationship with HCPs; differences between healthcare systems and cultures; and systemic barriers such as lack of interpreters.^25^ Similar to this paper, the review of 51 studies demonstrated barriers were multilevel; the majority of themes related to the joint-level factors, while limited studies showed individual-level constraints.

With respect to ‘offers and resistance’ this paper refutes an existing study of service users’ experiences of virtual maternity care in the UK during the pandemic, where authors found no data supporting this Candidacy construct i.e. no participants refused or resisted the care they were offered.^22^ A key difference between the two studies is the time frame of data collection, wherein our study included experiences across the duration of the pandemic (May 2022–February 2023) while theirs included data from March to September 2020, covering only the first 7 months of the pandemic which were characterised by acute crises and lack of information and guidance. This raises an interesting question about whether service users’ acceptance of care, when it was perceived to be less-than-optimal, and particularly in the way it was delivered during a health-system shock, decreased over time. In the early period of the pandemic, there was a sense of camaraderie and support from the community with support for frontline workers who were risking their lives to continue providing medical care, motivated by social campaigns such as ‘Clap for our Carers’.^29^ However, these feelings of solidarity may have diminished as the pandemic continued, and circumstances got worse.

We found women and partners had to increasingly advocate for themselves in order to receive the care they wanted, some who felt they were not able to do so for fear of being reprimanded and treated poorly, some feeling abandoned by the healthcare system. The restrictions on birth partners or additional people who could advocate on their behalf further compounded this problem, which has been reported in other work.^30^ Similar to our work, others have found human rights not being upheld and HCPs’ behaviour to be a barrier that prevented access.^31^ Our participants also described experiencing a ‘postcode lottery’ of maternity services^32^ characterised by significant discrepancies in quality and availability of services between different NHS Trusts and hospitals, in some cases making it harder for them to access high-quality care close to home. As such, service users, who were financially able, reported turning to private maternity services to supplement routine NHS care, such as additional ultrasound scans and postpartum lactation consultations. Researchers have found women choosing private midwifery care for ‘peace of mind’ and continuity of care, which were unavailable through public services,^33^ similar to our findings. This poses a real problem of pushing out women from lower socio-economic groups who may not be able to pay for private care, thereby widening pre-existing health inequalities.^34^

Our findings show a multilevel approach is necessary to tackle existing inequalities in access and experiences of maternity as prioritised in the Women’s Health Strategy for England.^35^ NHS maternity services were found to be increasingly difficult to navigate during the pandemic due to confusing and ever-changing guidance which led to widespread uncertainty. Other research supports our findings, also finding women resorted to obtaining information from friends and family instead^5^ due to limited confidence in HCPs.^36^ Ensuring clear and cohesive information and guidance will be instrumental in fostering trust between service users and HCPs and consequently enabling positive experiences and access, in planning for future health-system shocks. A personalised approach to implementation of digital modalities of care should be considered, taking into consideration each individual’s capacity to participate as it increases flexibility and access for some, but has the potential to exacerbate inequalities and digital exclusion for others.^22^ While our study also found a lack of cultural humility and competency among HCPs, it is important to acknowledge that amidst widespread pandemic-related service configurations, most HCPs were overworked, under-resourced and traumatised, making it extremely difficult to prioritise nuanced, person-centred care.^37^ The maternity workforce needs to be supported routinely with improved and widely available training and development that prioritises culturally competent care. Such programmes in the UK and other European countries have been viewed favourably to improve midwives’ knowledge, skills and self-perceived cultural competency.^38^ Finally, underpinning all future planning of maternity services is co-production with service users and local stakeholders,^39^ along with leveraging local organisational strengths through Maternity Voices Partnerships.^40^ However, MVP representation remains variable across the country and has limited ability to capture the voice of those hidden from the system, such as many migrant or homeless women.^41^ This calls for the need to strengthen inclusive and equitable engagement in co-production and better representation of underserved communities.

### Strengths, limitations and future directions

This study benefitted from a large and diverse participant sample of women and partners, a significant proportion belonging to ethnic minorities, high deprivation status, representation from gender and sexual minorities, and people with social complexity. We were able to include perspectives from across the UK, including the devolved nations, where guidelines were different from those in England. However, it is important to note individuals who participate in research are more likely to be early adopters and generally have a positive, or at least neutral, perception of healthcare.^42 43^ These reflections may not be shared by those who distrust the NHS and do not attend or seek care, unless absolutely necessary. We did not capture specific demographic information on housing precarity, insecure immigration status, caregiving burden and severe social risk such as domestic violence or incarceration, choosing instead to have participants self-identify social complexity. Although some of these social risk factors (e.g. migration status, caregiving burden) were discussed by participants during interviews, we may have inadvertently excluded those with the greatest barriers. By having a long period of recruitment, we gathered data on experiences across the pandemic lockdowns and when restrictions had been relaxed, including full implementation of the vaccine. Methodologically, analysing qualitative data through the theoretical framework of Candidacy allowed us to investigate multi-faceted and system-level factors that impact service users’ experience and eligibility for care. Furthermore, by testing the two new constructs of the extended Candidacy framework, we were able to assess the validity of these constructs in empirical research, which proved to hold true. However, as we did not specifically ask about participants’ immigration status or its impact on healthcare utilisation, data were limited, providing opportunities for future research.

## Conclusions

Maternity service reconfigurations implemented for safety during health-system shocks have significantly impacted women and their partners’ experience and ability to access care; and thus, should be carefully considered in future planning of equitable maternity services. Our study suggests that a multi-level approach which considers individual, health system and joint-level factors would be most impactful to garner change. By exploring the perspectives of both women and partners from a range of backgrounds across the UK, we have identified a set of overlapping and multifaceted barriers that need to be addressed in policy and practice to improve access to and experience of care for people with complex social and medical risk factors.

## Supplementary material

10.1136/bmjph-2025-004303 online supplemental file 1
