# Supplementary material for: Experiences of seeking and receiving maternity care during a health-system shock: a qualitative study with women and partners in the UK with a focus on marginalised groups
Source: BMJ Public Health. 2026 Mar 27;4(1):e004303. doi: 10.1136/bmjph-2025-004303 (PMC13034225; doi:10.1136/bmjph-2025-004303)
Supplement: online supplemental file 1 [file bmjph-4-1-s001.docx]

# **Supplemental file 1**

# **Post-Pandemic Planning: Interview Topic Guide- Women**

Thank you for taking the time to be interviewed in relation to this RESILIENT project. You are one of the women who received maternity care during the pandemic.

[Short blurb- This project is investigating how to plan for future maternity services in this country so that we are prepared for future health system shocks or another pandemic. Along with a quantitative and policy aspects of the project, we are speaking with mothers, partners, healthcare workers and policymakers who were involved with maternity care during the past 2 years to learn from their experiences and see what worked well, what needs to change and how we move forward]

I’d like to ask you a few questions to understand your experience with maternity services during the pandemic, and how you think we can move forward to provide the best maternity care to women. While this will require some reflection of experiences over the time since the pandemic began in Feb 2020, **we want to ensure that such reflections are brief, and the focus is on how we move forward in the best way possible.**

The interview will be structured in six parts: (1) Personal experience, (2) Information sharing, (3) Virtual care, (4) Vaccines (5) Ethics, and (6) Looking forward

We are interested in the full range of your experiences- there are no right or wrong answers, and you will not be judged based on what you say.

The interview will take approximately 30 mins–1hr.

We can provide you with the transcript, should you wish.

Should you feel uncomfortable at any time and wish to stop the interview, or take a break, please tell me.

Do you have any questions before we begin? If not, I shall now start to record.

*Just for the recording you are participant #XXX*

*Confirm DOD and check if they have had any other pregnancies during the pandemic.*

## **Section 1: Personal Experience**

1. **Can you tell me about your experience being pregnant and giving birth during the pandemic?**
2. **Were you able to have your partner (or someone else) there for support during your appointments and delivery? How did this make you feel?**
3. **Could you tell me any changes to your care that you received during the pandemic?**
   1. How does it differ to what you had expected your pregnancy care experience to be like?
   2. How did this make you feel?
4. Did you have COVID at any point during your pregnancy/during birth/first few weeks postnatal?
5. How was your experience with breastfeeding?
   1. Explore availability of formula, breastfeeding support etc.

**Section 2: Information Sharing**

1. **How was information relayed to you?**

Probes:

- - Format? Detailed guidelines/ Quick reference guide/executive summary/ Infographic

1. **Did you get all the information you wanted on changes in service provision/caring for you and your baby/ being safe from COVID-19?**

Probes:

- Did you have to do your own research?
- Was information easily accessible?
- What websites did you use?

1. **Did you understand everything? Was there an opportunity for you to raise questions? Were they answered in full?**
2. **How did you feel about the government’s guidelines for pregnant women during the pandemic such as shielding?**

## **Section 3: Virtual Care & self-monitoring**

My next set of questions will address virtual care and women’s self-monitoring of particular health problems.

1. **Did you receive any virtual maternity services yourself?**

Probes:

- - What form- telephone consultations/videoconferences?

1. **Did you have to perform any self-monitoring during your pregnancy?**

Probes:

- 1. Who gave equipment? Any checks?

1. **If you had to do any monitoring yourself during your pregnancy, what types of costs did you have to bear yourself?**

Probes:

- First probe on out-of-pocket expenses:
  - Did you buy anything to do with the monitoring, for example did you buy a BP machine or proteinuria strips or other (like a bathroom scale to weigh yourself)?
  - Was there anything else you bought?
- Second probe on time costs:
  - On what types of activities did you spend your time on related to your monitoring?
  - Did you experience any time lost from work or lower productivity related to your monitoring?

1. **Can you describe what worked well (or not) in receiving virtual care? in self-monitoring?**

Probes:

- - For telephone consultations/videoconferences, were there any technological challenges for you or the care providers? Was digital poverty a problem- did you have easy access to the internet and a device?
  - Is your first language English? If not, how did you manage this? Was it successful?

1. **How did receiving care virtually affect your day-to-day schedule?**

Probes:

- Was it easier (or not) to participate in your care?
- Easier (or not) caring for other children/ other commitments?

1. **How has receiving care virtually impacted your experience of the maternity care?**

Probes:

- - Did you feel like you were being cared for in the way you wanted?
  - Did you have any fears or concerns about the effectiveness or safety of virtual care (vs. face-to-face care) or self-monitoring?
  - Was there any impact on continuity of care, newborn care, outpatient visits, tertiary services & surgery?

1. **What types of costs have you had related to virtual care?**

- First probe on out-of-pocket expenses:

For example, did you have any of the following expenses:

- Transport (public transport or private car/petrol/parking) in case you had to travel somewhere else to join the virtual meeting
- Child care so you could join the virtual meeting
- Related to internet and connectivity – any data or telephone charges you had because of your virtual meetings
- Was there anything else you spent money on due to the virtual care you received?
- Second probe on time costs:
- On what types of activities did you spend your time on related to your virtual care?
  - - Did you experience any time lost from work or lower productivity related to your virtual care?

## **Section 4: Vaccine Hesitancy**

I’d like to now talk about the COVID-19 vaccine.

1. **What are your views about COVID-19 vaccination for women who are planning pregnancy, pregnant, or postpartum?**
2. **In terms of maternity health care providers, what are your views on mandatory vaccination? Is this something that’s important you with face-to-face appointments/delivery?**

Probes:

- - On what are these views based? (explore: RCOG? RCM? UK government? Other?)
  - Your views on public messaging (initially non-committal and then reassuring)?
  - Was information available easily? Was it clear? Did it balance known risks of COVID-19 with unknown, theoretical risks of vaccination?
  - Do you have any ongoing doubts?
  - Is there anything you would have liked to see but couldn’t find?

1. **If you chose to get vaccinated, what types of costs did you have?**

- First probe on out-of-pocket expenses:

For example, did you have any of the following expenses:

- Transport (public transport or private car/petrol/parking) to get vaccinated
- Child care so you could get vaccinated
- Was there anything else you spent money on due to getting vaccinated?
- Second probe on time costs:
- On what types of activities did you spend your time on related to getting vaccinated?
  - - Did you experience any time lost from work or lower productivity related to getting vaccinated?

## **Section 5: Ethical Framework**

I would now like to ask you to reflect on how care was provided for pregnant and postnatal women like you in the UK over the course of the pandemic.

1. **What are your thoughts on the care you and others like you received/ were treated?**
2. **What do you think morally and ethically?**
3. **What changes were/were not justified?**
4. **How did the way your look, your race/ethnicity, sexual orientation impact your care?**

Probes:

- Explore Q2/3 based on response to Q1- specific ethical opinion on changes; how things changed in height on pandemic vs. later

## **Section 6: Final Reflections & Looking forward**

My final questions are going to ask you to reflect on your personal experiences of receiving care in the pandemic and how you might envisage the future

1. **In your opinion, how would like for maternity services to be delivered in a future pandemic or other health system shock?**

Probes:

- - Amend the service
  - 'Hybrid’ of service provision practices from before and during the pandemic?
  - Are there practices that will/should go back to normal? Why?
  - Are there changes that will/should be stay? Why?
  - Do you have any *new* ideas for positive change?
  - Agree with vaccination of pregnant/postpartum women outside maternity services?
  - Do you believe these opinions are similar to the women?

1. **How do you imagine our best future for maternity services?**
   - 1. for yourself as an individual and your family?
     2. for the healthcare community?
2. **Do have any advice for any one in your situation having a baby at a time like this?**
3. **Do you have any other reflections that you feel would be important for us to hear as we imagine our ‘best future’ for the care of mothers and babies?**

*[Stop recording]*
